# Prevention of gastric cancer by *Helicobacter pylori* eradication: A review from Japan

**DOI:** 10.1002/cam4.2277

**Published:** 2019-05-23

**Authors:** Yoshiharu Uno

**Affiliations:** ^1^ Office Uno Column Kakogawa Japan

**Keywords:** atrophic gastritis, dysbiosis, gastric cancer, gastric intestinal metaplasia, *Helicobacter pylori* eradication, latent gastric cancer, proton pump inhibitors

## Abstract

Japan introduced a *Helicobacter pylori* eradication therapy strategy in 2013, with the aim of decreasing the number of gastric cancer‐related death, the number of new cases of gastric cancer, and associated medical costs. Five years have passed since then, but no reduction in the annual number of gastric cancer has been observed. In addition, it was suggested that the number of deaths due to gastric cancer could be reduced to 30,000 a year by 2020, but the annual death toll in 2017 remained at more than 45,000. Based on the above evidence, it was examined whether it was possible to reach the target value based on the data from the last 5 years. The number of deaths per year in 2020 is predicted to be more than 40,000, which is clearly different from the target value. Logically, the effect of the strategy might appear by 2023. However, there is a possibility that the risk of gastric cancer may increase in some populations due to the influence of proton pump inhibitors and dysbiosis in the gastric microbiome. To solve these problems, combined therapy with PPIs and aspirin for patients after *H pylori* eradication should be considered.

## 1 INTRODUCTION

Gastric cancer is clearly associated with infection by *Helicobacter pylori*, and in populations without *H pylori* infection*,* the gastric cancer incidence rates are very low.^1^ This fact led to the development of a strategy to prevent gastric cancer via the administration of *H pylori* eradication therapy. In Japan, the provision of eradication therapy for chronic gastritis due to *H pylori* infection by health insurance was started in February 2013, regardless of the presence of symptoms.^2^ The main purpose of this strategy was to reduce the number of gastric cancer‐related death and the number of new cases of gastric cancers. This strategy was believed to be economically beneficial because it would drastically reduce the cost of treating patients with gastric cancer within 5 years.^3^, ^4^ However, according to report published by the Ministry of Health, Labor and Welfare in January 2019, the number of new cases of gastric cancer in 2016 was 134 650, which was the largest number to date (Data S1).^5^ There was a 0.6% decrease in the number of deaths due to gastric cancer in 2016 compared to the previous year.^6^ One reason for the discrepancy between these results and the predictions may be that an insufficient number of people achieved eradication of *H pylori*. It is also possible that the strategy is ineffective. The purpose of this review was to provide evidence that it is impossible to attain the goal of the strategy and to explain the reasons for this failure.

## 2 A REALITY FAR FROM THE PREDICTED RESULTS

### 2.1 Theory underlying the strategy and the predicted number of gastric cancer‐related deaths

In Japan, owing to the baby boom that occurred between 1947 and 1949, the largest age group in the population is near 70 years. However, in Japan, the population naturally decreases due to various diseases after the age of 75 years. With the decrease in the prevalence of *H pylori* infection, the severity of atrophic gastritis and gastric intestinal metaplasia in the Japanese population has drastically decreased since 2010.^7^, ^8^ Furthermore, not only the rapid decline in *H pylori* infection rates after the baby boom but also the entire population is decreasing, and the total number of *H pylori*‐positive persons will also decrease after 2025 (Data S2). For that reason, it is clear that the number of deaths due to gastric cancer might naturally decline after 2025 even without the strategy to eliminate gastric cancer. Nonetheless, there is an urgent need for the eradication of *H pylori* to prevent gastric cancer in the elderly, including in baby boomers, who have a high risk of gastric cancer.^2^, ^3^, ^4^ This strategy was implemented to achieve a short‐term rather than a long‐term goal.

The specific predicted values were as follows.

#### 2.1.1 Predicted the number of new cases of gastric cancer

The basis for this prediction was the incidence of gastric intestinal metaplasia in Japan,^9^ and the prevention rate of metachronous gastric cancer (MGC) by *H pylori* eradication therapy.^10^ In Japan, the prevalence of gastric intestinal metaplasia in patients with *H pylori* was as follows: 51‐60 years, 47.5%; 61‐70 years, 57.6%; older than  71 years, 57.1%.^9^ In a study of gastric cancer reduction effect by *H pylori* eradication therapy on MGC in Japan, the incidence of secondary gastric cancer was reduced by approximately one‐third over 3 years.^10^ For subjects in this study, the prevalence of gastric intestinal metaplasia was approximately 50%.^10^ From the above, it was predicted that approximately 30% of gastric cancer would be suppressed by the eradication of *H pylori* in the group of over 50 years of age.^3^, ^4^ Asaka noted that the number of new cases of gastric cancer is 107,000 per year in Japan, therefore, 535,000 people develop gastric cancer in 5 years. However, if *H pylori* eradication therapy is performed for all people aged 50 years or older, the number of annual gastric cancers will be 32,000 (107,000 × 0.3). Therefore, within 5 years, the occurrence of 375,000 (535,000‐32,000 × 5) gastric cancers can be eliminated.^4^

#### 2.1.2 Predicted number of deaths

Because this strategy is intended to prevent the development of new gastric cancer, the number of patients dying from gastric cancer should decrease. Asaka and Kato described that if the gastric cancer elimination project is successful and about 50% of people with *H pylori* infection receive eradication therapy, the number of deaths from gastric cancer will be about 30,000 in 2020.^2^ Asaka described that this strategy could prevent approximately 150,000 deaths from gastric cancer over 5 years.^3^, ^4^ In fact, the calculation of the 2020 forecast value was made from the decrease in expected value in 5 years. Asaka explained that this strategy could prevent approximately 150,000 deaths from gastric cancer within 5 years. The number 150,000 was obtained assuming that 40% of people who developed gastric cancer would die (375,000 × 0.4).^4^ The hypothesis that the number of annual deaths would be 30,000 after 5 years was, first, assuming that the number of deaths in 5 years would be 70,000 if the strategy was not implemented.^3^, ^4^ Therefore, with a baseline of 50,000, the total reduction in 5 years is 100,000 (150,000−20,000 × 5 × 2^−1^). If half of the subjects receive eradication therapy, reduced numbers are 20,000 at 5 years later, and the annual number of deaths is 30,000 (50,000−20,000).

In Japan, the eradication success rates of first‐line treatment were about 90%, and most unsuccessful patients receive second‐line and third‐line treatment and, endoscopy is mandated before the first‐line treatment. However, the evaluation of the cost‐effectiveness of the strategy did not take into the cost of confirming that patients were *H pylori*‐negative after undergoing eradication therapy, the cost of endoscopies, or costs incurred by treatment failure. The cost‐effectiveness was evaluated based on the cost of the first test for *H pylori* and the cost of the first‐line treatment.^4^ Specifically, the eradication rate used in designing this strategy was based on the number of people undergoing first‐line treatment only.^4^ The number of subjects (42.266 million people) was based on 78% of the 54 million people over the age of 50 years being infected with *H pylori*. However, this infection rate is derived from data from 1990,^11^ and it is clearly different from the actual infection rate immediately prior to the implementation of the strategy. The use of this overly high *H pylori* morbidity rate may be one of the causes of the exaggeration of the predicted effectiveness of the strategy.

### 2.2 Results after implementing the strategy

**Figure 1:**
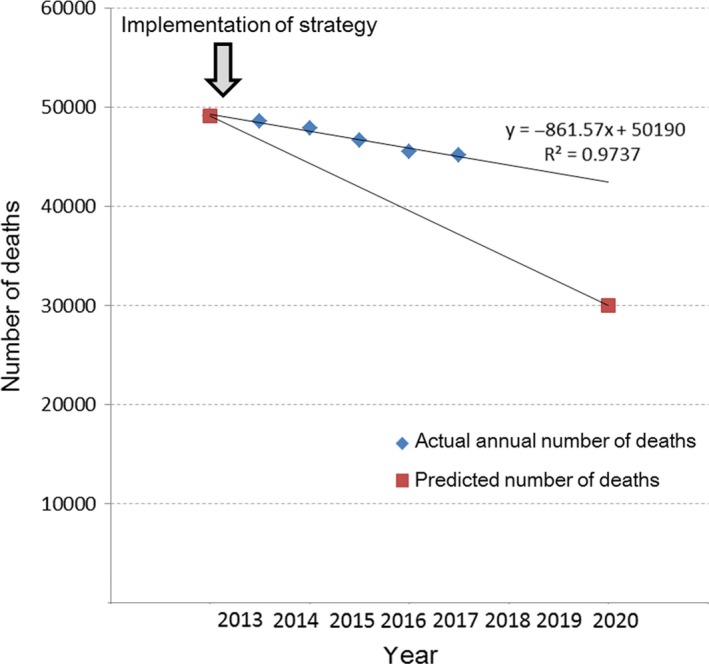
Predictive number before strategy and actual number after strategy. The number of deaths per year showed a very strong linear relationship (*R* ^2^ = 0.9737). According to extension of that straight line, even in 2020, the number of deaths from gastric cancer would over 40,000

The Japanese government approved the *H pylori* eradication therapy strategy for individuals with peptic ulcers in 2000 and further extended this therapy to every *H pylori*‐positive person in 2013.^2^ The adaptation of this eradication therapy covered all *H pylori*‐infected individuals, regardless of endoscopic/pathological findings, and became an indiscriminate eradication strategy. As a result, in Japan, 600,000 people annually from 2001 to 2013 and 1.4‐1.6 million people annually since 2013 have been administered *H pylori* eradication therapy,^12^, ^13^ and in total, 13.6 million people who were *H pylori*‐positive were treated between 2001 and 2016.^14^ Assuming that on average, 1.5 million people per year have received *H pylori* eradication therapy since the implementation of the expanded strategy in 2013, the total number of patients treated from 2013 to 2016 was 7.5 million, which is only 17.5% of the original target of 42.26 million people. However, by the time of the implementation of this strategy in 2013, the *H pylori* infection rate had decreased, and the number of *H pylori*‐positive people aged 50‐79 years was estimated to be 18.6 million people 15 (Data S3). Therefore, by 2016, approximately 40% of *H pylori*‐positive people in the 50‐79 year old age group may have already received *H pylori* eradication therapy. However, in general, gastric cancer screening in Japan has been expanded to include all individuals older than 40 years. Therefore, there are 22.2 million subjects undergoing gastric cancer screening, half (11.1 million people) of whom might receive *H pylori* eradication therapy prior to 2020. In the 5 years since the strategy was implemented, the number of deaths from gastric cancer has tended to decrease slightly (Data S4). However, according to the regression line (*R* ^2^ = 0.9737), the value predicted in 2020 is greater than 40,000, and the possibility of achieving that expected value is extremely low (Figure 1).

**Figure 2:**
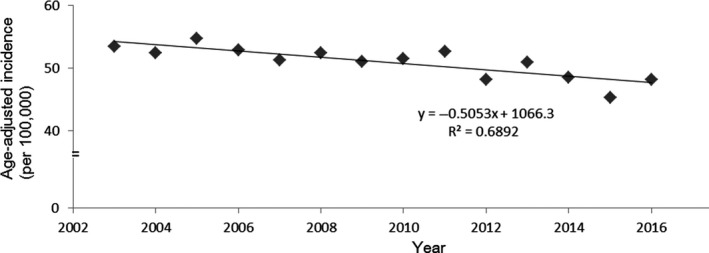
Age‐adjusted morbidity of gastric cancer from 2003 to 2016 in Japan. All data are on the regression line (y = −0.5053x + 1066.3, *R* ^2^ = 0.6892). Changes after 2013 cannot be confirmed

The reason for the difference between the predicted and actual deaths due to gastric cancer could be that gastric cancer incidence rate did not decrease as much as predicted. Clearly, the number of gastric cancer‐related deaths has decreased, although only slightly. However, the decrease in the number of deaths over 5 years has been approximately 10,000 people out of the 7.5 million who have received *H pylori* therapy, which is only 0.13% (Data S4). Although the number of gastric cancer sufferers is increasing, age‐adjusted morbidity rates from 2003 to 2016 have been declining gradually (Figure 2).^5^, ^16^ However, the rates after 2013 are also on the regression line (*R* ^2^ = 0.6892). These results suggest that *H pylori* eradication therapy could not have the effect of prevention of gastric cancer within 5 years. The increase in the number of affected individuals reflects Japan's population structure, and a gradual decline in age‐adjusted morbidity rates may reflect a natural decline in *H pylori* infection rates (Data S2).

## 3 CAN *H PYLORI* TREATMENT REDUCE THE INCIDENCE OF GASTRIC CANCER?

### 3.1 Latent gastric cancer after *H pylori* eradication

**Figure 3:**
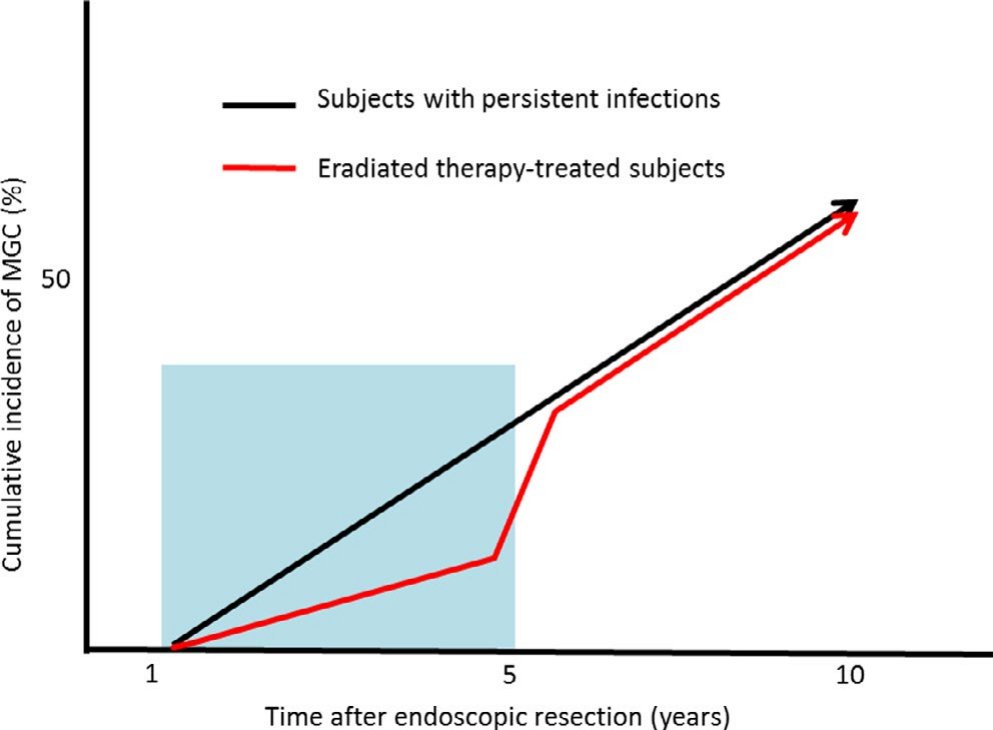
The effect of gastric cancer detection rate by latent gastric cancer. A short period of research within 5 years leads to erroneous results that *H pylori* eradication can prevent gastric cancer development

**Figure 4:**
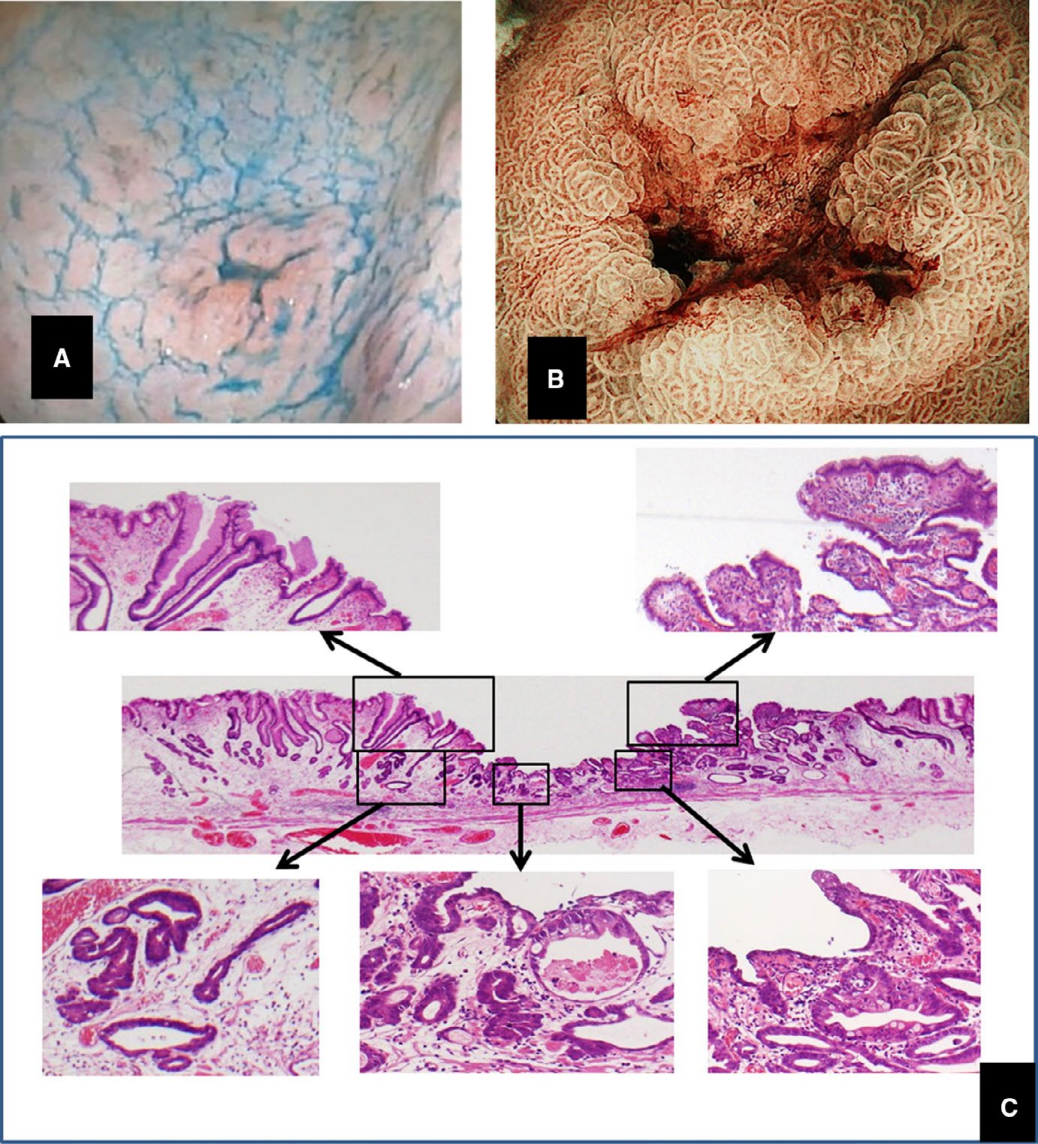
Typical early‐stage gastric cancer after *H pylori* eradication. A, Primary early‐stage gastric cancer found 3 years after *H pylori* eradication. B, The lesion removed by endoscopic submucosal dissection. The diameter of the depressed part is 3 mm. C, Carcinoma tissue is seen to be covered by nonneoplastic tissue

In this strategy, emphasis was placed on the reduction in the incidence of MGC to one‐third of the original rate in only 3 years.^2^, ^3^, ^4^, ^10^ This result led to the hypothesis that the occurrence of gastric cancer is suppressed immediately upon the eradication of *H pylori* and that the number of deaths due to gastric cancer could be drastically reduced within several years.^3^, ^4^ However, other research groups in Japan have reported that the eradication of *H pylori* does not prevent the occurrence of MGC.^17^, ^18^ In those studies, the incidence of MGC increased 4 to 5 years after *H pylori* eradication. In 2017, Maehata et al investigated why gastric cancer became more prevalent during the second half of the 11‐year study period.^19^ They observed that the gastric cancer that occurred within 5 years of eradication was morphologically characterized by small depressions covered with nonneoplastic mucosa. Because this finding differs from the early‐stage gastric cancer (EGC), which occurs in patients with conventional atrophic gastritis, it may be overlooked and classified as latent gastric cancer if the endoscopist is unable to recognize it (Figure 3).^20^ It has also been reported that the incidence of primary EGC increases 5 years after *H pylori* has been eradicated.^21^ Moreover, this new form has been observed not only in MGC but also in primary EGC after *H pylori* eradication (Figure 4).^22^, ^23^, ^24^, ^25^

Because this form of EGC is different from that of gastric cancer that occurs in the conventional *H pylori*‐infected stomach, there is a possibility that this form of cancer may be misdiagnosed or diagnosed after it has progressed to a later stage. In addition, the belief among endoscopists that gastric cancer is less likely to develop after *H pylori* eradication may lead to a detection bias. Various gastric mucosal changes (eg, mottled patchy erythema) occur after the eradication of *H pylori*.^26^ However, because there is no such change in the gastric mucosa without *H pylori* treatment, changes due to carcinogenesis can be easily detected by endoscopy. These factors may lead to differences in cancer detection rates.

### 3.2 Logical lead time from the eradication of *H pylori* to a reduction in the mortality from gastric cancer

At a certain point, level of intestinal metaplasia in the Correa pathway reaches the “point of no return”, which might limit of the efficacy of *H pylori* eradication for preventing gastric cancer.^27^, ^28^ Residual DNA methylation in the gastric mucosa after *H pylori* eradication plays a role in gastric carcinogenesis. This “point of no return” is closely related to the residual DNA methylation level.^29^ In a meta‐analysis of 10 studies performed in 2016, a reduction in the risk of gastric cancer due to *H pylori* eradication was not observed when considering the stages of intestinal metaplasia and dysplasia.^30^ In 2017, it was found that it takes 5‐10 years after *H pylori* eradication for atrophic gastritis to histologically improve to level found in uninfected subjects.^31^ Further, in another report from 2018, according to a 5‐year follow‐up survey of 1,755 cases after *H pylori* eradication, gastric cancer occurred in 5% of OLGA stage III and IV cases. Furthermore, 53% of stage II cases did not change, 19% progressed to stage III, and only 28% returned to stage 0 or I.^32^ These recent reports show that even in patients with gastritis in treatment‐responsive stages, the recovery time is long, and there are certainly cases that still progress to gastric cancer even after *H pylori* eradication.

**Figure 5:**
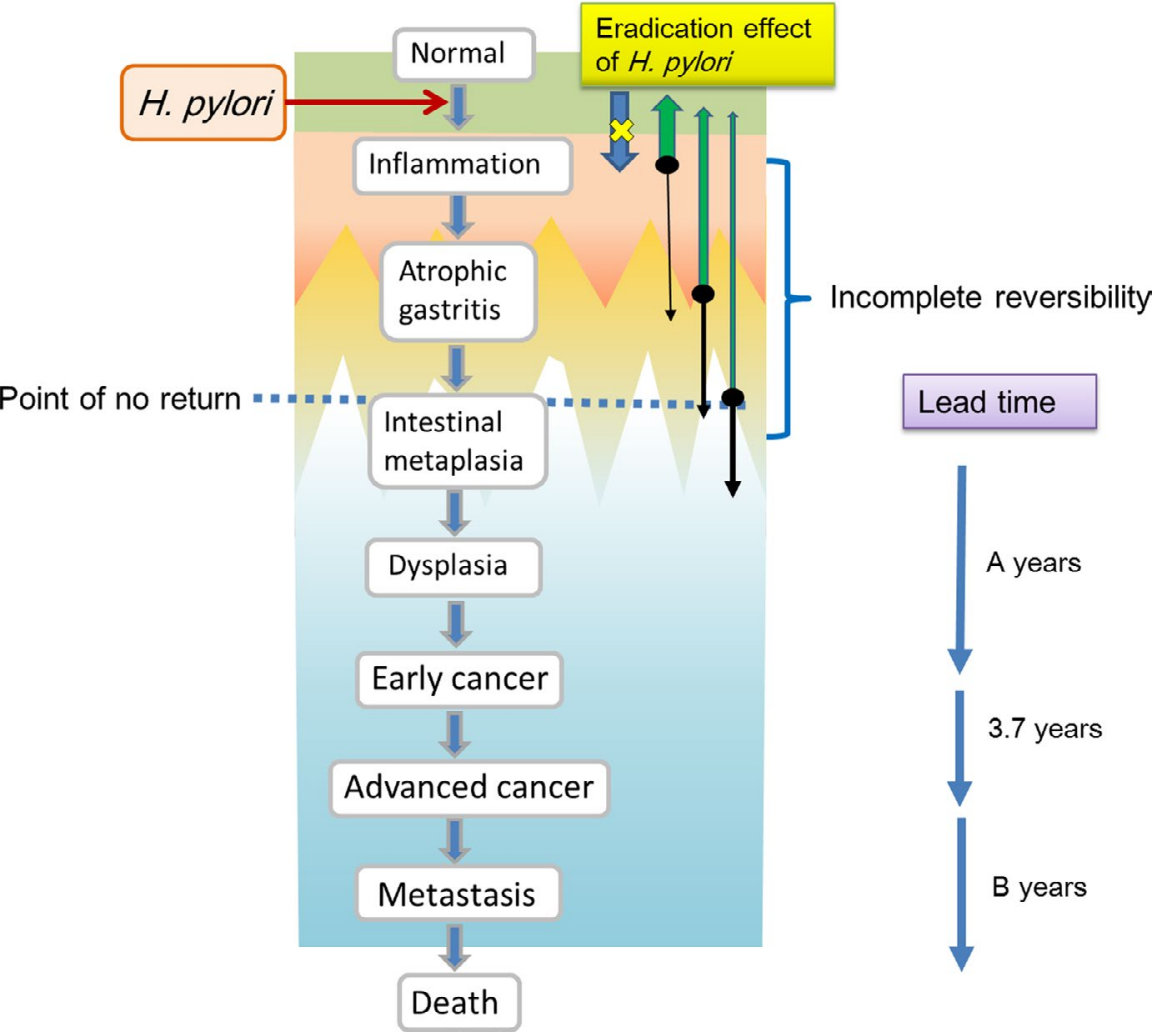
Lead time to the reduction in gastric cancer deaths from eradicating *H pylori.* After decreasing of gastric cancer which occurs from over stage of the "point of no return", the effect of *H pylori* eradication might be observed

When discussing the incidence of gastric cancer, it is necessary to consider the pathological criteria for diagnosing EGC. The mismatch in the pathological diagnoses of gastric cancer between Japan and the West has become a problem; lesions diagnosed as high‐grade dysplasia in the West are frequently classified as carcinoma in Japan.^33^, ^34^ However, lesions diagnosed as EGC based on Japanese diagnostic criteria have been demonstrated to progress to advanced cancer. In a Japanese study that followed  EGC discovered in 1980,^35^ the median of *Kaplan‐Meier* until the early cancer progresses to advanced cancer was 44 months (interquartile range [IQR]: 30‐80), cumulative 5‐year risk for progression to the advanced stage was estimated as 63.0% (95% confidence interval 48.1‐77.9％). Furthermore, the median until the death of delayed operation cases was 125 months (IQR = 45‐195). Almost all those EGCs may have originated from *H pylori* infections because the rate of *H pylori* positivity in the 1980s was estimated to be 70%‐80% in Japan.^7^ Considering the lead time from the development of atrophic gastritis or intestinal metaplasia to progression to EGC (A) and the lead time from the development of advanced cancer to death (B), in theory, it would take more than 3.7+A+B years to observe the effect of eradicating *H pylori* on gastric cancer‐related deaths (Figure 5). Based on the above evidence, an observable effect of *H pylori* eradication on gastric cancer‐related death cannot be expected within 5 years of the implementation of the strategy, although it might be possible to observe an effect within 10 years. However, even 10 years after the eradication of *H pylori*, the incidence rate of gastric cancer remains equivalent to the rate between 5 and 9 years after the administration of therapy, and permanent follow‐up is necessary.^21^ Leung et al divided the subjects who received *H pylori* eradication therapy into three groups of less than 40 years, 40‐59 years, and 60 years or older, and investigated the incidence rates of gastric cancer, and the incidence rates were compared in three periods (less than 5 years, 5‐9 years, and 10 years or longer) for the three groups.^36^ As for the results, although the incidences of gastric cancer in the *H pylori*‐treated cohort were comparable to the age‐matched general population in the first 9 years after eradication, after 10 or more years of *H pylori* treatment, the risk of gastric cancer among the older (≥60 years) and middle (40‐59 years) age groups became significantly lower than that of the matched general population. This evidence indicates that gastric cancer incidence and mortality cannot be reduced less than 9 years after the initiation of eradication therapy but may be reduced after 10 years.

#### 3.2.1 Influence of proton pump inhibitors (PPIs)

It has been suggested that the incidence of reflux esophagitis may increase after *H pylori* eradication in Asians, who demonstrate marked increases in gastric acid secretion after *H pylori* eradication.^37^ In a large‐scale cross‐sectional study of Japanese subjects, the prevalence of reflex esophagitis in subjects with chronic *H pylori* infection and successful *H pylori* eradication were significantly different (2.3% and 8.8%; *P* < 0.0001).^38^ A report from Taiwan stated that eradication of *H pylori* increased the reflux esophagitis morbidity rate from 13.7% to 27.3%.^39^ A researcher from South Korea reported that compared with the prevalence of reflux esophagitis in the persistent infection group, the prevalence of reflux esophagitis increased after successful *H pylori* eradication (OR 2.34).^40^ In addition, there is a possibility that PPIs will be used for the treatment of reflux esophagitis. However, the use of PPIs has been reported to increase the risk of gastric cancer.^41^, ^42^ Regarding causality between PPIs and gastric cancer, sample size, observation period, and sampling bias when gastric cancer is not confirmed before administration of PPIs, the involvement of potential drugs involved in gastric cancer development, such as aspirin, affects the research results.^43^ However, if gastric cancer increases in patients using PPI after *H pylori* eradication, the direct relationship between *H pylori* and PPIs might be denied. Furthermore, if the endoscopy is performed before *H pylori* eradication, sampling bias may be eliminated. In a cohort study in Hong Kong with 63,397 subjects, it was observed that 153 patients (0.24%) developed gastric cancer during a median follow‐up period of 7.6 years, and PPIs use was associated with an increased gastric cancer risk (HR 2.44).^44^ Long‐term use of PPIs was still associated with an increased gastric cancer risk in subjects even after the administration of *H pylori* eradication therapy. From the above evidence, it can be concluded that the prevalence of gastric cancer due to the use of PPIs may increase in certain populations after the eradication of *H pylori*. However, the risk of PPIs for gastric cancer might be avoided using a combination with aspirin. From the results of the cohort study, Cheung et al suggested that aspirin may be effective in preventing gastric cancer after *H pylori* eradication.^45^, ^46^ Interestingly, they found that gastric cancer risk reduction with aspirin in *H pylori*‐eradicated subjects (HR 0.30 [95% CI: 0.15‐0.61]) was greater than that reported in the meta‐analysis (pooled odds ratio [OR] 0.78 [95% CI: 0.69‐0.87]) including studies on both *H pylori*‐infected and *H pylori*‐negative subjects.^45^ The prevention effect on gastric cancer was more prominent in those who used aspirin daily or for 5 or more years. Furthermore, it has been reported that PPIs and aspirin combination therapy may prevent esophageal adenocarcinoma in Barrett's eosophagus.^47^ Concerning the relationship between aspirin and events of gastrointestinal bleeding, Garcia Rodriguez et al reported that concomitant use of PPIs and aspirin decreased upper gastrointestinal bleeding risks relative to aspirin monotherapy.^48^ From the above, it is expected to perform a randomized prospective study on whether combined therapy with PPIs and aspirin for patients after *H pylori* eradication can prevent gastric cancer and/or esophageal adenocarcinoma.

### 3.3 Gastric cancer and stomach microbiota

**Figure 6:**
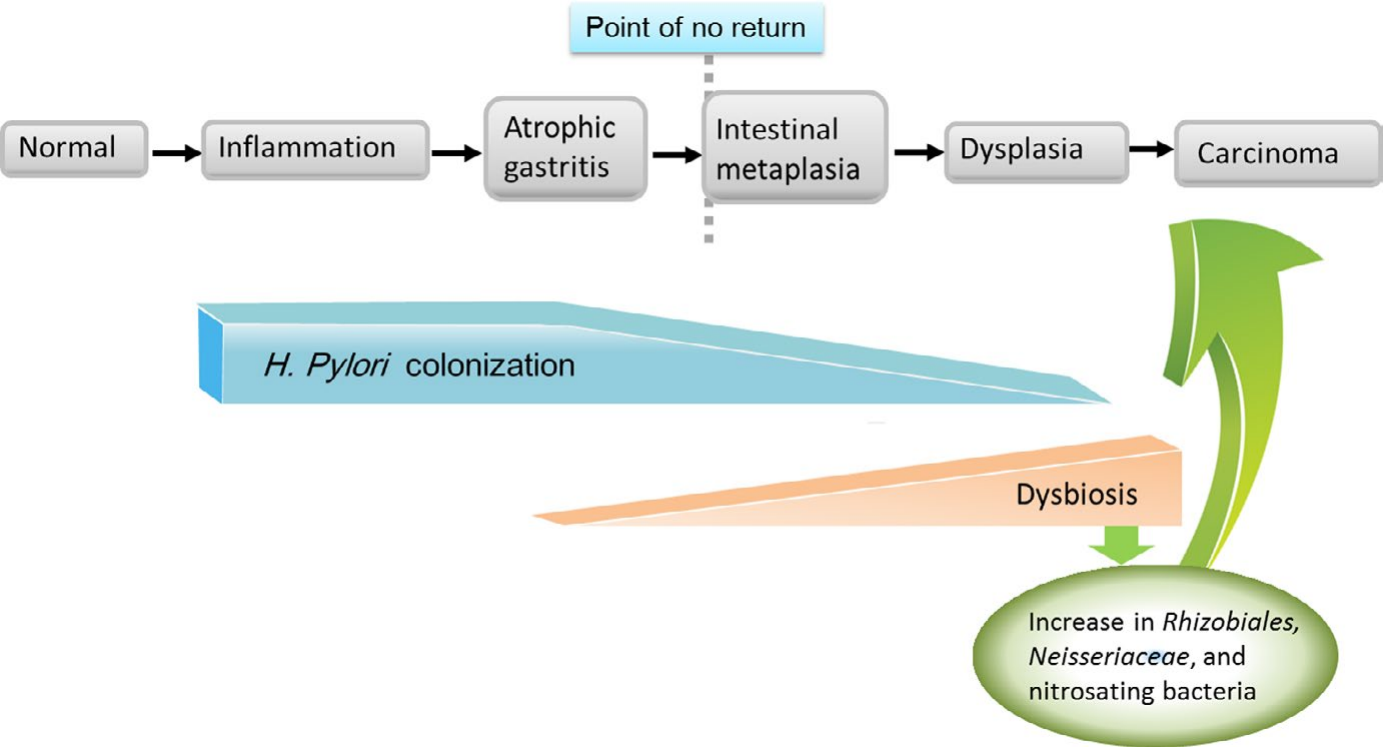
Relationship between stomach microbiota and gastric cancer

As atrophic gastritis progresses, *H pylori* itself spontaneously disappears from the stomach.^49^ However, this condition leads to the highest risk of developing gastric cancer.^50^^,^ 51 According to the gastric microbiota profile generated by 16S rRNA analysis, many other bacteria exist in the stomach besides *H pylori*.^52^ Chinese researchers confirmed that *H pylori* was less common in the tumoral microhabitat than nontumoral microhabitats by conducting a gastric microbiota analysis targeting the 16S rRNA gene in 230 normal, 247 peritumoral and 229 tumoral tissues.^53^ *H pylori* colonization alone is not sufficient to induce gastric carcinogenesis, and *H pylori*‐induced gastric cancer is promoted by the presence of a complex microbiota.^53^ Furthermore, dysbiosis of the microbiome in the stomach, which is involved in the development of gastric cancer, occurs when the relative abundance of *H pylori* decreases.^54^ Research from Portugal suggested that the gastric microbiome in patients with gastric carcinoma exhibits a dysbiotic microbial community (eg, nitrosating bacteria) with genotoxic potential.^55^ (Figure 6) Researchers from South Korea reported that the gastric microbiome in the successful *H pylori* eradication group resembled that in the *H pylori*‐negative intestinal metaplasia group, which is regarded as a high‐risk group.^56^ Studies of the gastric microbiome, such as these, may provide new strategies to prevent gastric cancer in the future.

## 4 CONCLUSION

The Japanese strategy of *H pylori* eradication therapy is unlikely to be able to achieve the predicted reduction in the number of gastric cancer‐related deaths based on the actual data from the last 5 years. Logically, the effect of the strategy might appear in 2023. However, there is a possibility that the risk of developing gastric cancer may increase in some populations due to the influence of PPIs and dysbiosis in the gastric microbiome. To solve these problems, combined therapy with PPIs and aspirin for patients after *H pylori* eradication should be considered.

## CONFLICT OF INTEREST

Uno Y declares no conflicts of interest in relation to the publication of this article.

## AUTHOR CONTRIBUTIONS

Uno Y contributed to the writing of the manuscript, creation of theory, reference collection, and creation of the figures.
