# Supplementary material for: Prevention of gastric cancer by Helicobacter pylori eradication: A review from Japan
Source: Cancer Med. 2019 May 23;8(8):3992–4000. doi: 10.1002/cam4.2277 (PMC6639173; doi:10.1002/cam4.2277)

**Supporting Information 1**

The chronological transition of the number of new cases of gastric cancer


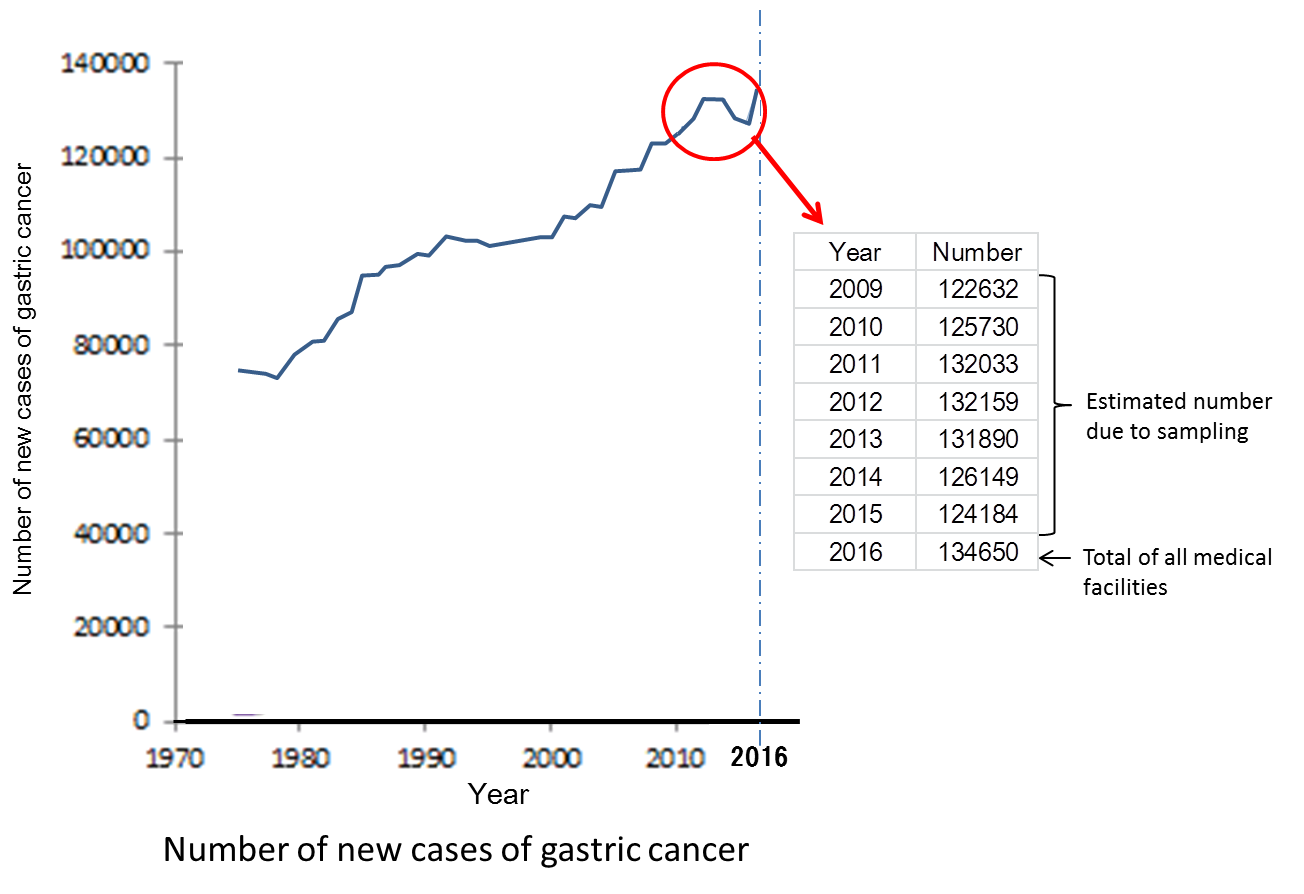


The annual number was an estimated number until 2015, but in 2016 the exact number was revealed by the National Cancer Registry Act.

Dara of 2016

Japan Health, Labor and Welfare Ministry. Result of national cancer registration in 2016.

<https://www.mhlw.go.jp/content/10900000/000468976.pdf>

Data of 2015

National Cancer Center Research Institute Japan

<https://www.ncc.go.jp/jp/information/pr_release/2019/20190117/index.html>

Datas of 2001-2014

National Cancer Center Research Institute Japan

<https://ganjoho.jp/reg_stat/statistics/brochure/monitoring.html>

Datas of 1975-2014

National Cancer Center Research Institute Japan

<http://gdb.ganjoho.jp/graph_db/gdb1?smTypes=5>


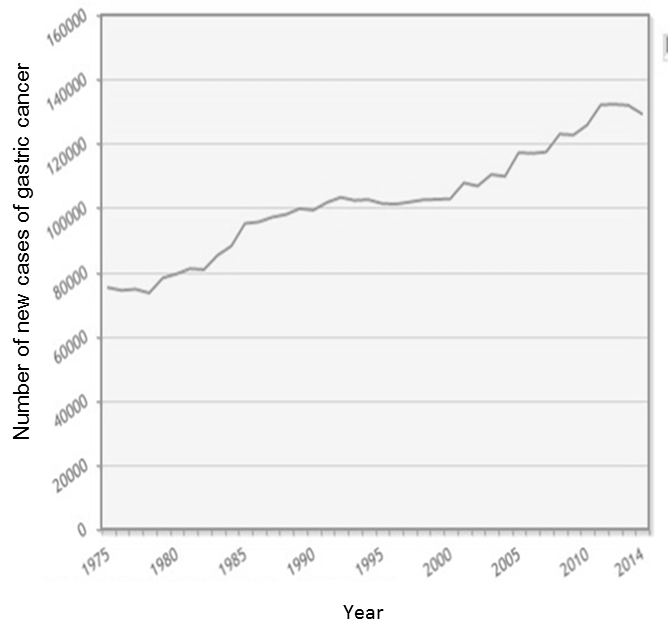

Supplement: Supplementary file 1 [file CAM4-8-3992-s001.docx]
