# Supplementary material for: Prevention of gastric cancer by Helicobacter pylori eradication: A review from Japan
Source: Cancer Med. 2019 May 23;8(8):3992–4000. doi: 10.1002/cam4.2277 (PMC6639173; doi:10.1002/cam4.2277)
Supplement: Supplementary file 2 [file CAM4-8-3992-s002.docx]

**Supporting Information 2**

Changes in the number of *H. pylori*-positive individuals in Japan


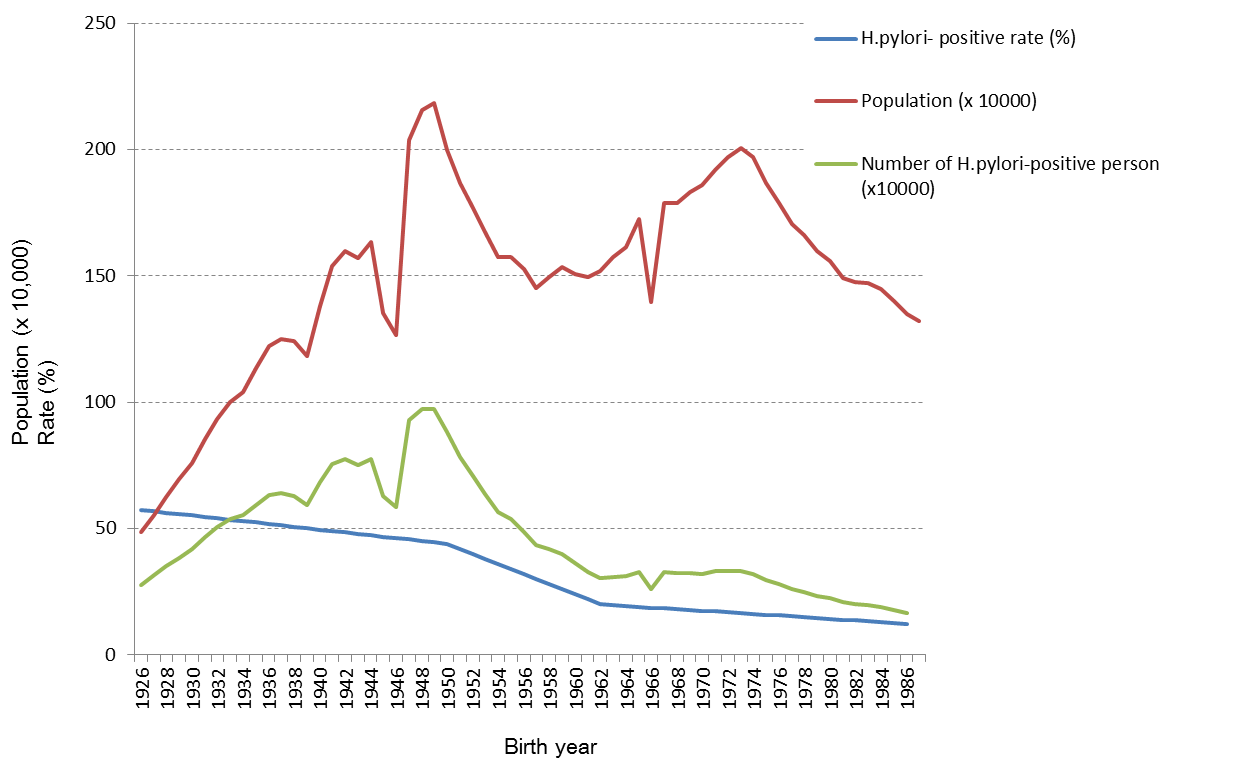


After the baby boom, with the rapid decline in *H. pylori* infection rate, the total number of *H. pylori*-infected people should decrease.

References

Japanese Statistics Bureau of the Ministry of Internal Affairs and Communications, the results of the 2015 census.

<http://www.stat.go.jp/data/kokusei/2015/kekka.htm>

Watanabe M, Ito H, Hosono S, *et al*. Declining trends in prevalence of Helicobacter pylori infection by birth-year in a Japanese population. Cancer Sci. 2015;106:1738–1743.
