# Supplementary material for: Prevention of gastric cancer by Helicobacter pylori eradication: A review from Japan
Source: Cancer Med. 2019 May 23;8(8):3992–4000. doi: 10.1002/cam4.2277 (PMC6639173; doi:10.1002/cam4.2277)
Supplement: Supplementary file 3 [file CAM4-8-3992-s003.docx]

**Supporting Information 3**

The prevalence of *H. pylori* infection was obtained from three regression lines of Watanabe et al., which is the prevalence of the median of each age group (20% at 45 years old, 27% at 55 years old, 43% at 65 years old, 48% at 75 years old.


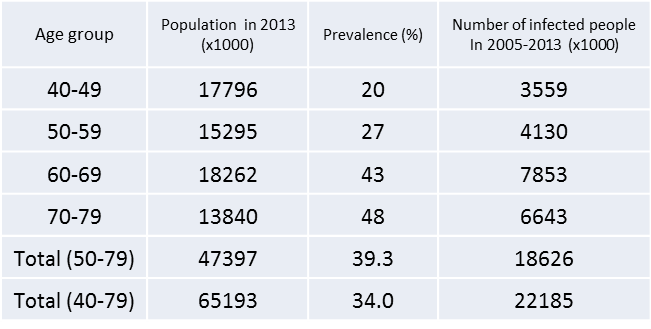


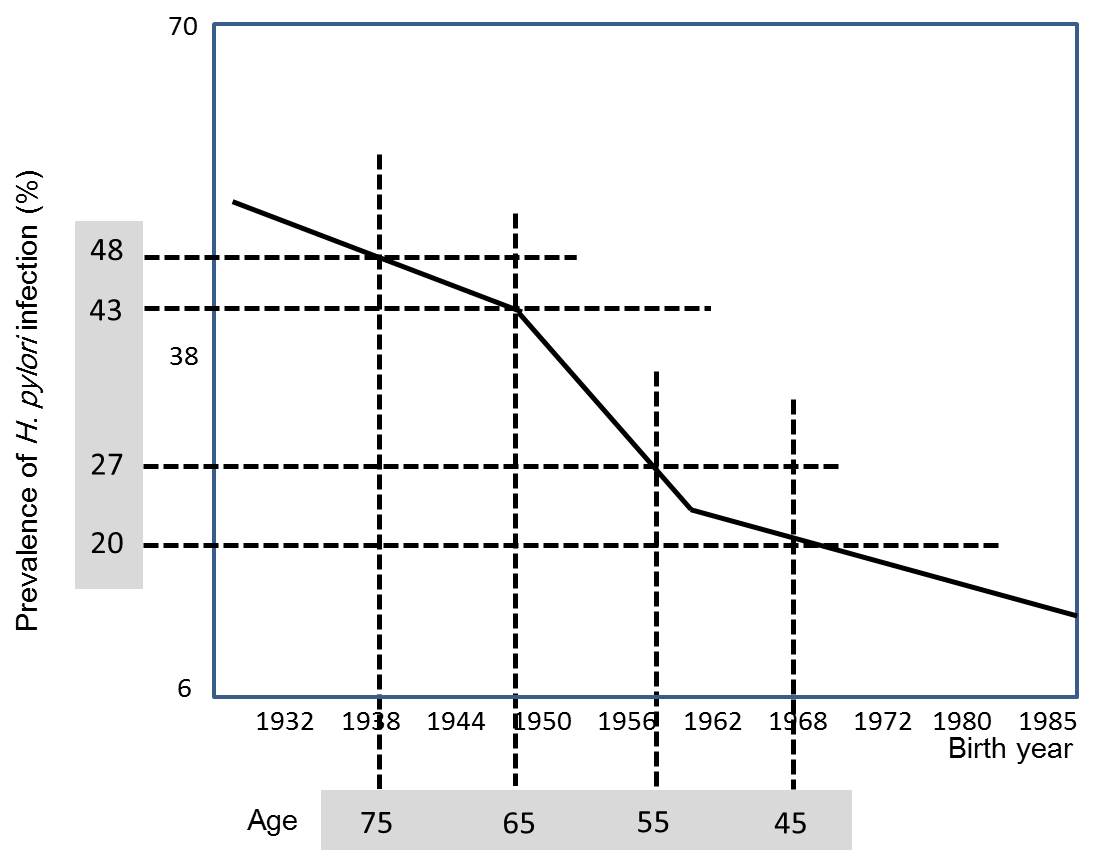


Reference

Watanabe M, Ito H, Hosono S, *et al*. Declining trends in prevalence of Helicobacter pylori infection by birth-year in a Japanese population. Cancer Sci. 2015;106:1738–1743.
