# Supplementary material for: Prevention of gastric cancer by Helicobacter pylori eradication: A review from Japan
Source: Cancer Med. 2019 May 23;8(8):3992–4000. doi: 10.1002/cam4.2277 (PMC6639173; doi:10.1002/cam4.2277)

**Supporting Information 4**

The annual number of deaths due to gastric cancer in Japan


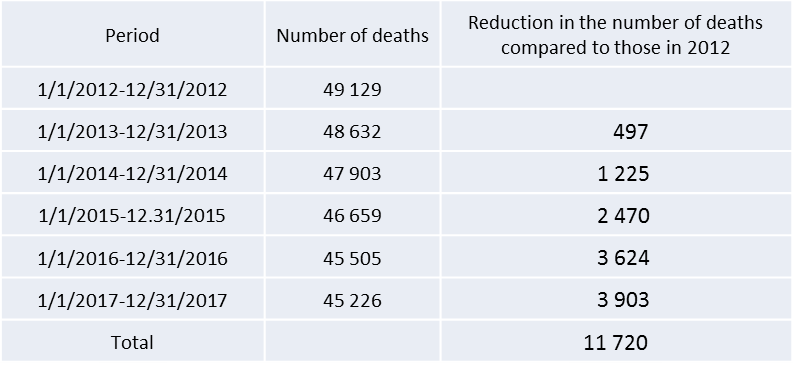


Datas of 1958-2017

National Cancer Center Research Institute Japan

<http://gdb.ganjoho.jp/graph_db/gdb4?dataType=20>


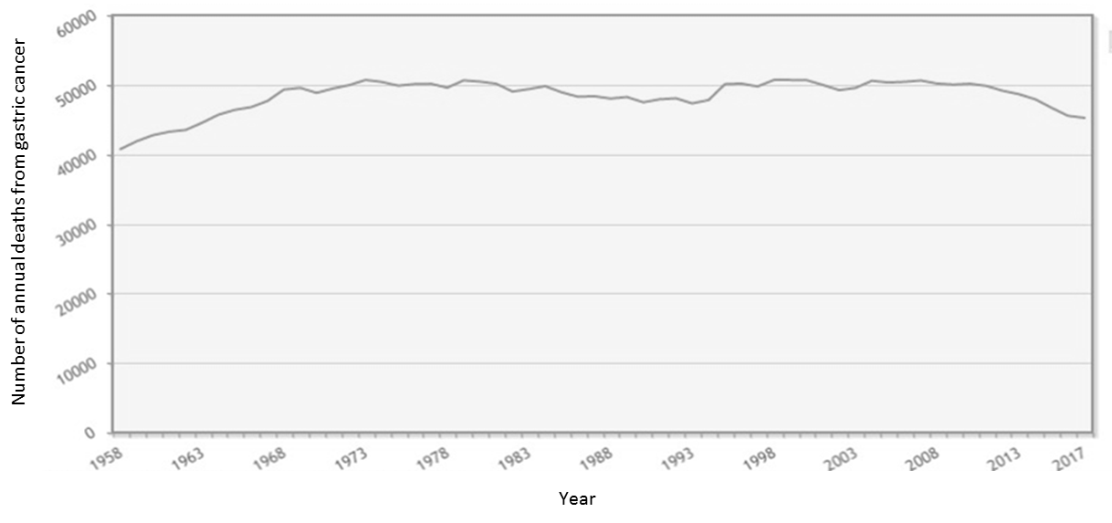

Supplement: Supplementary file 4 [file CAM4-8-3992-s004.docx]
